# THE IMPACTS OF VIRTUAL REALITY (VR) INTERVENTION ON DEMENTIA CARE EDUCATION: A SYSTEMATIC REVIEW

**DOI:** 10.1093/geroni/igae098.2984

**Published:** 2024-12-31

**Authors:** Xiaoli Li, Sandra Collins, Xiaoyu Liu, Ramesh Mutthina, Chung-Yi Chiu

**Affiliations:** Southern Illinois University Carbondale, Cabondale, Illinois, United States; Southern Illinois University Carbondale, Cabondale, Illinois, United States; Saint Louis University, St. Louis, Missouri, United States; Southern Illinois University Carbondale, Carbondale, Illinois, United States; University of Illinois Urbana-Champaign, Champaign, Illinois, United States

## Abstract

VR technology has been explored for its potential to deepen the understanding of individuals living with dementia, allowing students and caregivers to immerse themselves in the lives of those affected by this condition. To date, research evidence regarding the implementation and effectiveness of VR interventions in this domain have not been evaluated and synthesized. We aimed to address this gap in the evidence. The study protocol was registered with PROSPERO, and literature published from 2000 onwards was searched from six databases: Academic Search Complete, APA PsycINFO, CINAHL, MEDLINE, Web of Science, and PubMed. Additionally, hand searches of keywords and citation tracking were conducted in Google Scholar. Both qualitative and quantitative literature were considered, and study reporting quality was assessed using the JBI Critical Appraisal Checklists. Among the 318 articles initially identified, 22 were selected for the review. The review encompassed 17 experimental studies and five qualitative studies, involving formal / informal caregivers and university students. Sample sizes ranged from 20 to 278 participants, originating from seven countries and regions, utilizing 11 distinct VR prevention programs. Overall, the incorporation of VR in dementia care education emerges as a novel and underexplored research avenue. VR interventions demonstrated enhancements in caregivers’ and students’ empathy, knowledge, attitude, and understanding of people with dementia, although not all experimental studies found statistically significant changes. VR-based education emerged as a valuable complement to traditional teaching and training methodologies, offering a more engaging and realistic learning experience. However, further research in larger and more diverse samples is needed.

